# Perceived organizational politics and employees’ innovative behavior: a cross-level joint moderation model

**DOI:** 10.1038/s41598-026-59802-2

**Published:** 2026-07-06

**Authors:** Xiaona Xu, Jingxue Wang, Yan Liu

**Affiliations:** School of Economics and Management, Shenyang Aerospace University, No. 37 Daoyi South Street, Shenyang, 110136 Liaoning People’s Republic of China

**Keywords:** Perception of organizational politics, Political skill, Environmental uncertainty, Employees’ innovative behaviour, Joint moderating effect, Business and management, Business and management, Politics and international relations, Psychology, Psychology

## Abstract

Although prior research has linked perception of organizational politics (POP) to employees’ innovative behavior (EIB), the cross-level boundary conditions under which political skill and environmental uncertainty jointly shape this relationship remain underexplored. This study investigates the impact of POP on EIB, and introduces political skill (at the individual level) and environmental uncertainty (at the organizational level) to further examine the cross-level boundary conditions, i.e., the interactive effects of individual-level and organizational-level factors. Based on an empirical study of 402 samples from 39 Chinese companies, the results indicated that the POP was negatively related to EIB, and political skill negatively moderated the relations between POP and EIB. Moreover, political skill better reduced the negative impact of POP on EIB in organizations with higher environmental uncertainty. Our findings uncover the constraining mechanism of POP on EIB from a cross-level perspective, focusing specifically on how political skill and environmental uncertainty jointly moderate this relationship. The aim is to uncover the interaction pattern between individual and organizational factors within a behavioral science framework, rather than offering prescriptive managerial guidelines. This study accordingly extends the theoretical scope of research on organizational politics and environmental uncertainty.

**Supplementary Information:**

The online version contains supplementary material available at https://doi.org/10.1038/s41598-026-59802-2.

## Introduction

The significance of innovation in enhancing an organization’s long-term survival and competitiveness is widely acknowledged under the pressures of increasing global competition and a dynamic and volatile operating environment^1^. Certainly, employees are vital for driving organizational innovation, and employees’ innovative behavior (EIB) facilitates bottom-up innovation^2^ and helps firms translate individual creativity into sustained competitive advantages^3^. Given this, along with powerful evidence that innovation positively affects organizational performance, investigating the antecedents of EIB is crucial^4^.

Rooted in Confucian traditions, Chinese organizations generally prioritize hierarchy, authority and interpersonal bonds. However, these cultural characteristics are not the direct measurement variables of this study. Normally, in their daily interactions, organizational members jointly and creatively generate and apply new thoughts or solutions to problems solving. They are also accustomed to including emotional factors in their judgement, thinking and behavior. These culture-driven workplace characteristics distinguish organizational dynamics in China from typical practices in Western organizational contexts, such as paying attention to guanxi and the pursuit of moderation. Organizational politics is an inherent, unavoidable feature of organizational life^5,6^. Driven by limited resources and conflicting interests, political behaviors among individuals and work groups are prevalent in organizations, leading to certain political behaviors or strategies used by all levels to pursue the maximization of their own interests^7^. Organizational members’ subjective cognitions and evaluations of the individual, group and organizational pursuit of self-interest are regarded as the perception of organizational politics (POP)^5^. It serves as a key perceptual lens through which employees interpret daily workplace interactions in the Chinese cultural context.

The cornerstone of innovation lies in ideas, and individuals are the core subjects that develop, advance, respond to, and revise ideas^8^. Accordingly, identifying factors that facilitate or inhibit EIB carries critical theoretical and practical significance^9^. Workplace EIB has attracted substantial scholarly attention in prior research^4,10^. A broad set of individual and organizational determinants have been verified to predict EIB, including leadership styles^1,2,9,11^, organizational innovation climate^12^, perceived organizational support^13^, employees work engagement^4^, developmental job experience^10^, high-involvement work practices^14^, psychological safety^3^, employees well-being^15^, digital human resource management (digital-HRM)^16^, and employees perceptions of high-performance work systems^17^. Recent scholarship further confirms that individual dispositional traits (e.g., proactive personality) positively shape EIB via work-related flow^18^. Person-environment fit theory also elaborates how individual attributes interact with organizational contexts to motivate EIB.

However, the predictive effects of several established EIB antecedents remain equivocal, leaving notable research gaps in this research domain^13^. Specifically, POP has been consistently documented to constrain employees innovative capacity^19,20^, yet two critical limitations weaken current understandings of the POP–EIB linkage. First, most existing studies adopt a single-level moderating design confined merely to the individual level, failing to adopt cross-level analyses to unpack the interactive effects of individual- and organizational-level factors on this relationship. Second, even if organizational-level variables are incorporated, prior empirical work predominantly applies single-level statistical approaches instead of multilevel modeling. Moreover, despite growing evidence highlighting the pivotal role of political skill in resource mobilization and innovation enactment^21^, scholarly insights into political skill in the POP–EIB nexus remain scarce. Prior studies generally regard political skill as a direct predictor of workplace outcomes, rather than a contingency moderator whose effects are contingent on organizational contextual features.

In addition, EIB is jointly shaped by four interconnected systems: individual attributes, leadership characteristics, team dynamics, and organizational innovation climate^9^. Innovation requires intensive cognitive and psychological resource investment from employees, as well as supportive contextual conditions^4^. Scholars have highlighted the necessity of exploring the interactive effects between organizational context and individual traits on employees’ innovation, given that contextual factors can either strengthen or weaken the association between individual characteristics and innovative outcomes^12^. Nevertheless, extant research tends to examine individual-level influential factors in isolation, with insufficient attention paid to the joint impacts of individual and organizational factors on the degree and form of employees’ innovation. Individual cognition and motivation, together with job characteristics and contextual elements, are validated pivotal predictors of employees’ innovation. Existing theories also note that organizational climate can redirect innovation-focused attention and behavioral engagement^22^. Even so, only a few EIB antecedent studies adopt a holistic perspective to unpack how individual traits interact with organizational environments to determine EIB. Within dynamic and complex organizational settings, both political skill and environmental uncertainty can reshape employees’ psychological states and behavioral responses^23,24^, which further alters employees’ innovative engagement.

To address the above research gaps, this study explores the POP–EIB relationship, and examines the cross-level joint moderating effects of organizational-level environmental uncertainty and individual-level political skill on this linkage. This study offers two key contributions to the extant literature. First, it fills the research gap regarding EIB formative factors, particularly political-based inhibitory factors. Second, it establishes a cross-level theoretical framework to clarify the boundary conditions that amplify or alleviate the POP–EIB relationship. Overall, the findings advance contextualized knowledge of organizational politics, environmental uncertainty and EIB within the Chinese cultural context, and broaden relevant research boundaries.

## Theoretical background and hypotheses

### Perception of organizational politics and employees’ innovative behavior

This study builds its theoretical model on social information processing theory (SIPT), the overarching theoretical framework of this research. According to SIPT, individuals form social cognition by interpreting organizational environmental cues and then adjust their attitudes and behaviors accordingly^25^. In this study, POP acts as a critical environmental cue. Political skill shapes individuals’ interpretation of these cues, while environmental uncertainty governs their salience and interpretive scope. This integrated perspective differs from prior studies that separately adopted psychological climate theory, social exchange theory or conservation of resources theory. It allows us to examine main effects, two-way moderation and three-way joint moderation within a unified logical framework. Furthermore, SIPT provides a consistent theoretical foundation for developing the research hypotheses.

Notably, psychological climate theory^26^ is a specific application of SIPT in organizational research, and the two theories are not mutually exclusive. Psychological climate theory highlights individuals’ cognitive perceptions of the environment, whereas SIPT explains how these perceptions translate into attitudes and behaviors. Our theoretical framework aligns with existing research on psychological climate and social exchange, while we adopt SIPT as the overarching integrative logic. Extant literature confirms that employees’ perception of organizational politics at work leads to adverse outcomes, including reduced organizational citizenship behavior^27^ and lower proactive performance^25^. Scholars have also explored its associations with employee creativity and innovation^19,20^.

As defined by Kwon and Kim, EIB refers to the generation and implementation of novel or revised ideas, processes, practices and policies^4^. Specifically, workplace EIB encompasses identifying problems and solutions, generating original ideas, garnering support for ideas, and implementing them in practice^28^. Scott and Bruce first proposed a three-stage model of innovation and developed the measurement scale for individual innovative behavior^9^. Later, Kleysen et al. classified individual innovative behaviors into five dimensions: opportunity exploration, generativity, preliminary investigation, championing, and application^29^. Based on these researches, EIB refers to employees’ voluntary generation and implementation of novel, valuable ideas, workflows and products at work.

High levels of POP discourage employees from engaging in innovative activities^25^. From the perspective of social exchange theory, the workplace functions as a social marketplace where employees strive to gain rewards through their efforts. Employees and organizations maintain reciprocal exchange obligations, a dynamic shaped by organizational culture and the political environment^30^. SIPT posits that individual behaviors are affected by peer conduct and situational factors. Organizational members develop social cognition based on environmental cues within the organization, which in turn shapes their attitudes and behaviors^25^. Employees who perceive supportive relationships with their organization tend to hold positive work attitudes and actively participate in extra-role behaviors to facilitate organizational success^6^, including innovative activities.

By contrast, in organizations characterized by a pervasive political climate, unfair and irrational practices render the effort-reward exchange relationship unpredictable. Perceived organizational unfairness undermines employees’ sense of obligation, resulting in poorer job performance and less favorable work attitudes^31^. Such negative perceptions toward the organization reduce employees’ intrinsic and extrinsic motivation for innovation, while heightening job anxiety and emotional distress^5^. These changes ultimately curb employees’ engagement in innovative work^25^.

In addition, organizational knowledge accumulation and sharing serve as the foundation for promoting EIB, which relies heavily on routine workplace interactions and communication. In highly political organizations, however, ambiguity in workplace interactions undermines employees’ psychological safety, which is not conducive to knowledge sharing and employees’ innovation^3^. Employees tend to be vigilant of others’ behaviors and suitably lower their interactions for avoiding troubles to secure their interests, reputation, and status. The defensive behavior of organizational members with high vigilance can lead to knowledge hiding^19^, further exacerbating the negative attitude of employees and reducing innovative behavior. In addition to destroying social exchange relationships and reducing psychological safety, POP can also indirectly hinder EIB by triggering knowledge-sharing hostility, such as knowledge hoarding and resistance to sharing ideas with colleagues^20^. This mechanism further verifies the negative impact of POP on EIB from the perspective of knowledge exchange. We thus propose:

#### H1

POP is negatively related to EIB.

### Moderating effect of political skill

Although the academic perspective on organizational politics is predominantly negative, political skill is regarded as a set of positive traits essential for individuals to effectively navigate their environments^32^. Political skill refers to the ability to accurately understand colleagues in the workplace and leverage such insights to influence others for the achievement of personal and organizational goals^33^. It comprises a broad set of social competencies. We argue that political skill reflects employees’ capacity to manage interpersonal dynamics and pursue social effectiveness. This capability can predict work attitudes and job performance. It also helps individuals obtain, accumulate and deploy scarce resources to adapt to diverse environments. By projecting confidence and sincerity, politically skilled employees can gain others’ trust and exert interpersonal influence^33^.

Extant research has documented a wide range of outcomes associated with political skill. For instance, it positively predicts job performance and entrepreneurial performance, and facilitates knowledge sharing and opportunity recognition, among other work and social outcomes^24,34,35^. A growing body of work has further examined its moderating roles across various criteria, including knowledge transfer, employee voice, affective commitment, organizational citizenship behavior and innovative behavior^6,21,36–38^.

Political skill is vital for regulating work attitudes and behaviors, as well as for adapting to changes in internal and external environments. It has therefore become an indispensable competency for interpersonal communication, information gathering, opportunity identification and resource allocation^34,38^. In challenging contexts, political skill enables employees to secure key resources to cope with adverse conditions such as organizational politics, and ultimately improve job performance^6^. Although POP exerts a negative influence on EIB, employees with strong political skill can effectively navigate politicized work settings. Consistent with this view, Kaur and Kang demonstrated that political skill weakens the impacts of organizational politics perceptions on knowledge hiding and organizational citizenship behavior^38^.

Individuals with high political skill firmly believe they can control the process and outcomes of social exchanges. They are able to accurately interpret peers’ intentions and use such understanding to influence others toward personal or collective objectives^35^. Equipped with strong social astuteness and interpersonal competence, they develop a thorough awareness of the workplace and colleagues. Accordingly, they can flexibly adjust communication strategies in light of intra-organizational power dynamics and political climates, and maximize the effectiveness of interpersonal interactions.

Taken together, the above arguments suggest that political skill mitigates the detrimental effect of perception of organizational politics on innovative behavior mainly by strengthening individuals’ perceived control over ambiguous political environments and boosting their social interaction efficacy. In turn, it alleviates psychological strain and withdrawal responses induced by political perceptions. In short, political skill does not eliminate organizational politics itself, but reshapes how individuals cognitively process politicized situations. Political skill fosters organizational identification and interpersonal trust^6^, thereby mitigating the inhibitory effect of POP on innovation. Similar to political skill, proactive personality, as another positive individual trait, can alleviate the negative impact of adverse organizational contexts on EIB by enhancing work-related flow^18^. This indicates that positive individual characteristics can promote innovative behaviors by improving psychological states, which supports the moderating role of political skill in the POP-EIB relationship.

In addition, existing literature has researched the positive impacts of political skill on employees’ psychological activities, such as improving work mood, releasing work pressure and reducing work rejection^24^. At the same time, positive work mood, low pressure and strain have a beneficial effect on improving innovative behavior^39^. Accordingly, we infer that employees possessing high political skill are likely to efficiently utilize their deep insights to recognize and use new knowledge, while obtaining relevant information, resources and other support from internal and external groups through communication, persuasion, negotiation and manipulation, which will promote resource exchange and improve job satisfaction. The construction of a harmonious interpersonal relationship network and the reduced work pressure will alleviate the inhibiting effect of the POP on innovation intentions, improving the enthusiasm for innovation in a situation characterized by low pressure and more sufficient resources. Since the role of political skill is to mitigate or even eliminate the negative effects of political situations^38^, we believe that high political skill will diminish the negative influence of the POP on EIB. We thus propose:

#### H2

Political skill negatively moderates the influence of POP on EIB.

### Joint moderating effect of political skill and environmental uncertainty

The enterprises today face higher uncertainty of external market and technological environment. Environmental uncertainty describes the complexity and unpredictability of an organization’s external market and technological environment as a circumstance wherein upcoming technology, markets, and other environments are difficult to foretell^40^. It is caused by the gap between the information needed for decision-making and the available information^41^. Because of the scarcity of pertinent environmental information and the limited access to process details, individuals cannot accurately evaluate and predict the changing status of the external environment, decision-making results and the trend of organizational development. Innovation is a behavior characterized by divergent thinking and problem solving. Some existing studies have linked it to the environment of the organization, showing the effect of environmental uncertainty on innovation^41,42^. An unstable external environment expedites strategic organizational modifications, augments the impact of dynamic capabilities, and fosters the emergence and proliferation of innovative behaviors.

According to Andrews et al., the link between political skill and job outcomes is contingent on factors that shift the direction or magnitude of its influence^43^. Among such factors, environmental uncertainty shapes the psychological states and work behaviors of both managers and employees^23^. An organizational environment is jointly shaped by objective conditions such as environmental uncertainty and leaders’ personal traits. For instance, leaders’ Dark Triad traits exert dual moderating effects on the association between challenge demands and EIB: they directly weaken this positive relationship, yet indirectly strengthen it by nurturing an entrepreneurial culture^44^. This evidence implies that the joint moderation of individual and organizational factors on EIB is far more complex than prior research has assumed.

Ferris et al., argued that employees with strong political skill are better able to adapt to highly dynamic settings, and that high environmental uncertainty also facilitates the effective deployment of political skill^33^. A key question thus arises: why does environmental uncertainty amplify the moderating impact of political skill? Theoretically, environmental uncertainty essentially reflects pervasive rule ambiguity and information asymmetry. In highly uncertain contexts, formal institutions and explicit regulations cannot address all emergent situations, which creates greater room for political skill to function. Specifically, rising uncertainty increases employees’ reliance on informal information, social support and interpersonal trust—core resources that political skill helps individuals secure effectively.

Accordingly, political skill exerts a stronger buffering effect against POP in highly uncertain organizations. By contrast, stable environments are governed by formal rules, which diminishes the marginal value of political skill. This reasoning re-conceptualizes the interplay between environmental uncertainty and political skill from a loosely associated pair to a context-dependent tool-environment fit. When employees can comprehend and manage their surrounding environment effectively, the negative impacts of POP are alleviated.

A highly dynamic environment brings organizational instability and persistent crisis risks. Under such circumstances, political skill serves as a vital instrument for acquiring and mobilizing innovation-related resources, which in turn fosters the emergence and development of innovative behavior. The underlying mechanism is as follows: greater environmental uncertainty expands rule vacuums and reshapes resource flows within organizations, offering broader scope for political skill—a competency centered on obtaining informal resources and building interpersonal trust. In stable environments, by comparison, formal rules and standard procedures prevail, leaving political skill with relatively low marginal utility.

While employees often build political connections to gain easier access to resources, political skill, as an effective approach to resource acquisition, allocation and coordination^6^, can be implemented more smoothly and yield better outcomes amid high uncertainty. Furthermore, contingency theory posits that individuals must adjust their behaviors to adapt to environmental changes and uncertainties^41^. High environmental uncertainty motivates employees to engage in interpersonal interactions and creates more opportunities for communication and collaboration. It also lowers resistance and raises tolerance toward political behaviors in the workplace.

In this scenario, employees proficient in political skill can build social networks to earn trust from others. They can also demonstrate sincerity to mitigate anxiety and pressure induced by POP, and remove barriers to innovative behavior. Notably, an alternative theoretical perspective exists: high uncertainty may make employees more defensive and risk-averse, and more inclined toward resource conservation. In this case, uncertainty would constrain rather than strengthen the functioning of political skill. To address these competing arguments, the present study incorporated both rationales in statistical analyses and empirically distinguished between them by examining the significance of the three-way interaction term. We thus propose:

#### H3

Environmental uncertainty strengthens the negative moderating effect of political skill on the POP-EIB relationship.

**Fig. 1:**
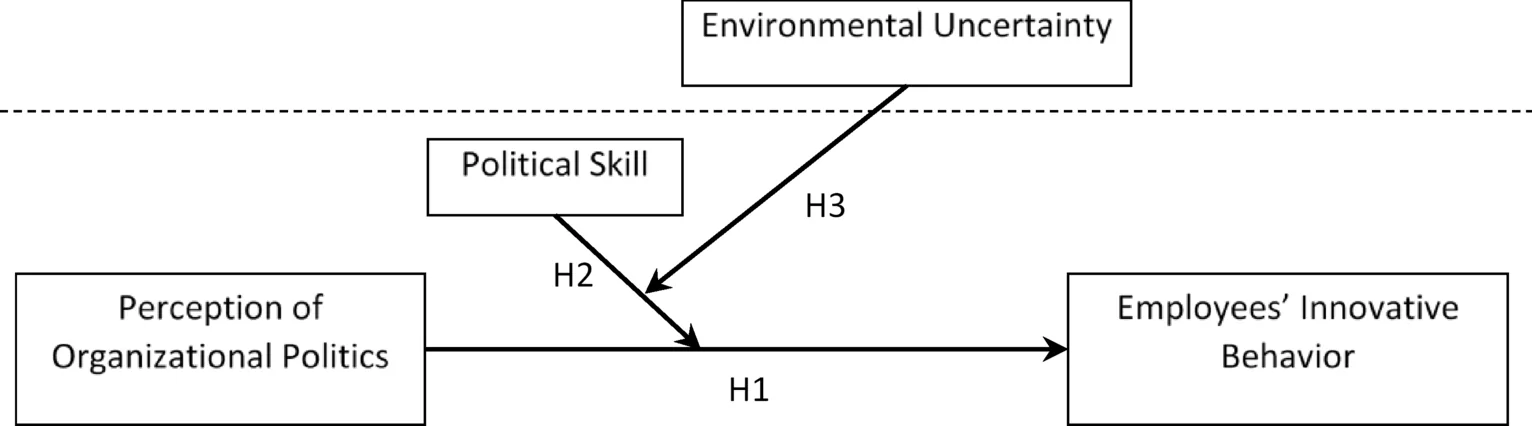
Theoretical model. This model proposes a theoretical framework for cross-level joint moderating effects. At the individual level, variables encompass perception of organizational politics (POP), political skill (PS), and employees’ innovative behavior (EIB), while at the organizational level, the variable is environmental uncertainty (EU). Hypothesis 1 posits that organizational political perception exerts a direct negative influence on employees’ innovative behavior. Hypothesis 2 suggests that political skill plays a negative moderating role in the relationship between organizational political perception and employees’ innovative behavior. Hypothesis 3 indicates that environmental uncertainty will strengthen the negative moderating effect of political skill (i.e., cross-level joint moderating effect). The direction of the arrow denotes the influence path among variables, and the solid line represents the relationship path of the core research hypothesis.

The theoretical model for this study is presented in Fig. 1.

## Methodology

### Sample and data collection

In the pre-survey stage, we distributed 108 questionnaires to MBA students who had been employed for at least 1 year, ensuring that they had experience with organizational politics and were familiar with the organizational environment. Although all scales used in this study were adapted from established measures, the pre-survey served two purposes: (1) to assess item clarity, readability, and cross-cultural appropriateness in the Chinese context, and (2) to identify any ambiguous or culturally inappropriate wording that might require minor adjustments. Based on feedback from the pre-survey, we made slight wording modifications to a few items to improve clarity, while keeping the core meaning of each scale unchanged. The formal questionnaire was then finalized.

Our formal sample consists of employees working in various departments of Chinese enterprises. We obtained institutional ethical approval and contacted firms via purposive sampling to solicit participation. Out of the contacted companies, 45 companies consented to attend this research. Data collection was from July to December 2024. To minimize common method biases, the independent and dependent variables were kept separate. During the first information collection stage (S1), demographic information and responses on POP, political skill and environmental uncertainty were collected. In this stage, questionnaires were given to 680 employees from 45 enterprises, and 543 (79.9%) were returned from 39 enterprises. One month after S1, the second stage of data collection (S2) for the EIB involved distributing 543 questionnaires with numbers to employees who participated in S1, and 475 (89.3%) were returned. After conducting data matching and filtering out incomplete responses, the final valid sample included 402 employees (59.1%) across 39 organizations, including junior staff and managers.

Sample sizes across groups ranged from 5 to 21 participants (mean = 10.3). In the cross-level analysis, POP and political skill at the individual level were group-mean centered, while environmental uncertainty at the organizational level was grand-mean centered. The model was specified as a random-intercept model, and no random slopes were estimated because the three-way interaction involved cross-level effects. The mean within-group agreement (Rwg) for environmental uncertainty was 0.867, with ICC(1) = 0.701 and ICC(2) = 0.957. The relatively high ICC(1) reflects substantial heterogeneity across industries (e.g., the coexistence of manufacturing and internet sectors), which is theoretically justifiable given that environmental uncertainty is an organizational-level perceptual construct.

### Measurements

Following the multi-method approaches and measurement design principles outlined by Gary, all constructs in this study were assessed using 5-point Likert scales (1 = strongly disagree to 5 = strongly agree)^45^ to ensure reliability and validity.

*Perception of organizational politics (POP)* Considering the significance of the indigenous characteristics of the POP and to align with the actual situation of Chinese enterprises and local research, we adopted Ma et al.’s 16-item scale^46^, which was adjusted and developed on the basis of Kacmar and Carlson’s scale^47^. The following is an example item: “In (our) organization, people should think carefully about who they can’t go against.”

*Employees’ innovative behavior (EIB)* To assess EIB, we utilized a 6-item scale developed by Scott and Bruce^17^. The following is an example item: “I search out new technologies, processes, techniques, and/or product ideas”.

*Political skill (PS)* We measured political skill using the 4-dimensional scale created by Ferris et al., which includes 18 items^33^. The following is an example item: “I spend a lot of time and effort at work networking with others”.

*Environmental uncertainty (EU)* Environmental uncertainty is measured via individual ratings and aggregated to the organizational level for cross-level analysis. The measurement of environmental uncertainty was based on the research of Miller, and it is measured in two dimensions with appropriate modifications^48^. Technical uncertainty and market uncertainty were measured by three items respectively. For example, “The speed of technology updates in the industry is very fast”, “Customers’ preferences and demands for products change rapidly”.

*Control variables* In the hierarchical regression analysis, we included control variables at both the individual and organizational levels. Individual-level controls included gender, age, educational level, tenure and position. Organizational-level controls consisted of enterprise ownership, enterprise size and industry, which are well-established predictors of EIB.

While the perceptions of organizational politics (POP) scale contains three subdimensions and the political skill (PS) scale comprises four subdimensions, this study focuses on the global constructs rather than individual subfacets. Consistent with the original scale validation studies^33,47^ and mainstream empirical practices, we adopted composite total scores for hypothesis testing. Supplementary second-order confirmatory factor analyses (CFAs) were conducted in “Scale analysis” section. The satisfactory model fit verifies that all subdimensions load onto a unified higher-order latent construct, which provides solid statistical justification for aggregating item scores into composite indicators. Thus, we used composite scores instead of second-order latent scores in multilevel regression analyses.

## Results

### Data aggregation testing

In this research, environmental uncertainty is an organizational-level variable, so it is necessary to evaluate the intra-group consistency (R_wg_) and inter-group differences (ICC) to reflect the consistency of the team members. According to the statistical results, the average within-group agreement (Rwg) for environmental uncertainty was 0.867. Combined with ICC(1) = 0.701 and ICC(2) = 0.957, all indices met the conventional thresholds for data aggregation. Therefore, it is appropriate for environmental uncertainty to be aggregated from individual-level data.

### Common method bias

We tried to mitigate the common method bias caused by the self-reported scale in two ways. In terms of programme control, the items were set in a concise and clear manner, and the respondents answered anonymously. The confidentiality rules were explained to the subjects, and their answers were indicated to be neither right nor wrong. The subjects were guided to answer according to their actual conditions to reduce the deviation caused by psychological pressure and expectation effects. Moreover, we conducted the Harman single factor test to control for statistical biases, which revealed that the first factor accounted for 30.342%, which met the standard of less than 50%. Additionally, the correlation coefficient of each variable was less than 0.6 (as shown in Table 3), meeting the standard of less than 0.9. Collectively, common method bias does not pose a serious threat to the current dataset. We also acknowledge that common method bias may not be completely eliminated, given the reliance on employees self-reports. Future studies could adopt supervisor evaluations of employees’ innovative behavior to further enhance the reliability of results through diversified assessment sources. Additionally, we utilized variance inflation factors (VIF) to assess multicollinearity and confirm the strength of the correlations between independent variables. The test revealed that the highest VIF value was 1.168 (< 5.0)^49^. Hence, there is no multicollinearity problem in our study. Overall, the threat of common method bias to the findings of this study remains within an acceptable range, though it has not been entirely eliminated. Given that employees’ innovative behavior continues to be assessed via self-reporting, there exists a potential for common source bias.

### Scale analysis

SPSS26.0 and MPLUS8.3 were used to analyse reliability and validity and to conduct the confirmatory factor analysis of the POP, EIB, political skill and environmental uncertainty. Regarding reliability, as demonstrated in Table 1, the Cronbach’s alpha coefficients of all variables were greater than 0.9, and C.R. values surpassed the minimum cutoff value at 0.6. In terms of validity, firstly, the AVE values ranged from 0.647 to 0.686, exceeding the benchmark of 0.5. Consequently, discriminant validity was supported. Additionally, all factor loadings exceeded the cutoff value of 0.5, confirming the convergent validity. Secondly, the structural validity of the multidimensional variables was verified. The results indicated that the fitting indexes of the POP (x^2^/df = 1.394 < 5, RMSEA = 0.031 < 0.1, CFI = 0.992 > 0.8, SRMR = 0.024 < 0.08), political skill (x^2^/df = 1.757 < 5, RMSEA = 0.043 < 0.1, CFI = 0.981 > 0.8, SRMR = 0.034 < 0.08) and environmental uncertainty (x^2^/df = 4.380 < 5, RMSEA = 0.092 < 0.1, CFI = 0.982 > 0.8, SRMR = 0.022 < 0.08) all met the standard. As a complement, Table 2 reports the results of a comparison among four-factor models using traditional single-level CFA. Although this model (x^2^/df = 3.609 < 5, RMSEA = 0.081 < 0.1, CFI = 0.810 > 0.8, SRMR = 0.071 < 0.08) did not meet the common threshold of 0.90, it was significantly superior to the alternative models, providing basic support for the discriminant validity of the constructs. In addition, the square root of the AVE for each latent variable was greater than the correlation coefficient between the variables (as shown in Table 3), also indicating good discriminant validity.

**Table 1:** Reliability and convergent validity analysis.

Measurement	FL	α	C.R	AVE
POP (POP1-POP16)	0.761–0.872	0.944	0.969	0.662
PS (PS1-PS18)	0.760–0.878	0.946	0.972	0.662
EU (EU1-EU6)	0.766–0.866	0.907	0.929	0.686
EIB (EIB1-EIB6)	0.731–0.892	0.914	0.916	0.647

POP, Perception of organizational politics; EIB, Employees’ innovative behaviour; PS, Political skill; EU, Environmental uncertainty.

**Table 2:** Comparison of measurement models.

Model	Factors	x^2^	df	x^2^/df	RMSEA	CFI	SRMR
Model 1	4 factors: POP, PS, EU, EIB	3547.180	983	3.609	0.081	0.810	0.071
Model 2	3 factors: POP, PS + EU, EIB	5025.604	986	5.097	0.101	0.700	0.111
Model 3	3 factors: POP + PS, EU, EIB	6675.974	986	6.771	0.120	0.577	0.143
Model 4	2 factors: POP + PS, EU + EIB	7941.437	988	8.038	0.132	0.484	0.159
Model 5	2 factors: POP + PS + EU, EIB	8144.727	988	8.244	0.134	0.469	0.162

POP, Perception of organizational politics; EIB, Employees’ innovative behaviour; PS, Political skill; EU, Environmental uncertainty.

**Table 3:** Means, standard deviations, and correlations (*n* = 402).

Variable	M	SD	POP	PS	EU	EIB
POP	2.371	0.882	**(0.814)**			
PS	2.763	0.879	− 0.342***	**(0.814)**		
EU	2.766	1.122	− 0.128*	− 0.098*	**(0.828)**	
EIB	3.675	0.932	− 0.342***	0.523***	0.310***	**(0.804)**

Significant values are in [bold].

Values in parentheses are the square roots of the AVE for the respective scales.

POP, Perception of organizational politics; EIB, Employees’ innovative behaviour; PS, Political skill; EU, Environmental uncertainty.

*Correlation is significant at the 0.05 level; **Correlation is significant at the 0.01 level; ***Correlation is significant at the 0.001 level.

Table 3 presents the descriptive statistics for the variables. As seen from the correlation coefficient, the POP is significantly negatively correlated with EIB (*r* = − 0.342, *p* < 0.001), which is consistent with the theoretical expectation and providing initial support for the research hypothesis. However, since other related factors are not controlled, regression analysis will be carried out in the following section. The significance of the correlation coefficients in Table 3 is based on two-tailed tests, with asterisks denoting conventional thresholds in psychological research (*p < 0.05, **p < 0.01, ***p < 0.001). All specific p-values output from SPSS26.0 have been verified to fall below their respective thresholds.

### Hypothesis testing

The hypothesis testing results using MPLUS8.3 are presented in Table 4. According to the regression results, POP exerted a significant negative effect on EIB (Table 4 M1: γ = − 0.107, *p* < 0.01). H1 was supported. To examine H2, the interaction effect of POP × PS on the EIB was included, and the results revealed a significant interaction effect (Table 4 M3: γ = 0.267, *p* < 0.001), proving that political skill has a significantly negative moderating effect on the relationship between the POP and EIB. H2 was supported.

**Table 4:** Hypothesis testing: multiple regressions.

Variables		Employees’ innovative behavior			
		M1	M2	M3	M4
Constant		3.544*** (0.508)	3.680*** (0.461)	3.703*** (0.425)	3.585*** (0.445)
Individual level	Gender	0.016 (0.035)	0.014 (0.036)	− 0.005 (0.036)	− 0.024 (0.033)
	Age	− 0.017 (0.028)	− 0.015 (0.028)	− 0.016 (0.024)	0.006 (0.024)
	Educational level	− 0.021 (0.022)	− 0.020 (0.023)	− 0.009 (0.023)	0.002 (0.023)
	Tenure	0.021 (0.029)	0.015 (0.029)	0.027 (0.027)	0.017 (0.027)
	Position	− 0.001 (0.027)	− 0.001 (0.027)	0.013 (0.024)	0.028 (0.024)
Organizational level	Enterprise ownership	− 0.087 (0.255)	− 0.097 (0.222)	− 0.096 (0.189)	− 0.031 (0.119)
	Enterprise size	− 0.136 (0.119)	− 0.130 (0.110)	− 0.104 (0.099)	0.039 (0.078)
	Industry	0.245* (0.103)	0.200* (0.098)	0.164 (0.090)	0.040 (0.080)
POP		− 0.107** (0.040)	− 0.106** (0.040)	− 0.098** (0.033)	− 0.118*** (0.033)
Individual level	PS		0.188 (0.137)	0.238 (0.140)	0.406** (0.152)
	POP × PS			0.267*** (0.074)	0.386*** (0.075)
Organizational level	EU				0.092 (0.177)
	POP × EU				0.038 (0.047)
	PS × EU				− 0.043 (0.130)
	POP × PS × EU				0.197** (0.071)
σ^2^		0.113*** (0.016)	0.114*** (0.016)	0.104*** (0.014)	0.085*** (0.013)
τ_00_		0.605*** (0.114)	0.509** (0.148)	0.396** (0.141)	0.134 (0.141)

POP and PS are computed as scale composite total scores; second-order CFA results in “Scale analysis” section confirm the existence of overarching latent constructs underlying their respective subdimensions.

POP, Perception of organizational politics; EIB, Employees’ innovative behaviour; PS, Political skill; EU, Environmental uncertainty.

*N* = 402; *Correlation is significant at the 0.05 level; **Correlation is significant at the 0.01 level; ***Correlation is significant at the 0.001 level.

**Fig. 2:**
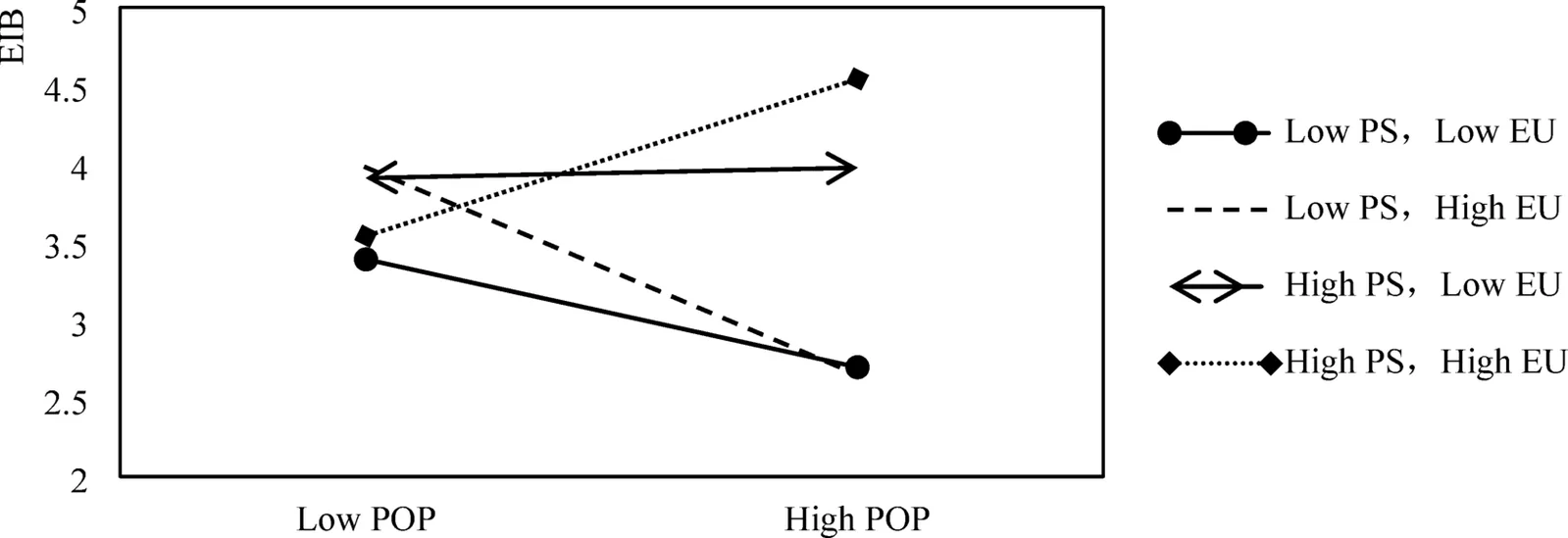
Influence of three-dimensional interaction on employees’ innovative behavior. This figure visually depicts the joint moderating effect of varying levels of political “skills (low or high) and environmental uncertainty (low or high) on the relationship between “organizational political perception (POP)-employees’ innovative behavior (EIB)”. The horizontal axis implicitly denotes the high and low levels of organizational political perception, while the vertical axis represents the predicted values of employees’ innovative behavior. The data annotations correspond to the specific predicted values of employees’ innovative behavior under different combinations of conditions. The findings indicate that in a context of high environmental uncertainty, the group with high political skills can more significantly mitigate the negative impact of high organizational political perception on employees’ innovative behavior compared to the group with low political skills. Nevertheless, in a context of low environmental uncertainty, the buffering effect of political skills is relatively feeble, thus validating the cross-level joint moderating mechanism of Hypothesis H3.

Furthermore, to test H3, the EIB was regressed on the three independent variables (i.e., POP, PS, and EU), their two-way interaction effects (i.e., POP × PS, POP × EU, and PS × EU), and the three-way interaction effect (i.e., POP × PS × EU). The results indicate that the three-way interaction effect on the EIB was significantly positive (Table 4 M4: γ = 0.197, *p* < 0.01). More intuitively, we plotted the moderating effects of political skill and environmental uncertainty in Fig. 2. The findings illustrate that within organizations with higher environmental uncertainty, political skill can better reduce the negative effect of POP on EIB.

## Discussion

This study explores how perception of organizational politics shapes employees’ innovative behavior. Based on existing research, POP has consistently been found to be a restriction of employees’ innovation^19,20^. However, the understanding of the boundary conditions of the POP-EIB remains underdeveloped and relatively few studies have taken a holistic view to explore whether and how the individual characteristics and interactions with the organizational environment affect EIB. Therefore, we examine the influence of the POP on EIB and introduce political skill and environmental uncertainty to further explore the cross-level boundary conditions. The findings indicate a significantly negative relationship between the POP and EIB, and political skill can reduce this negative effect. Moreover, environmental uncertainty significantly affects the moderating effect of political skill, that is, political skill can better reduce the negative impact of the POP on EIB in organizations with higher environmental uncertainty. We also found that the moderating effect coefficients of political skill (Table 4: M3: POP × PS, γ = 0.267, *p* < 0.001; M4: POP × PS × EU, γ = 0.197, *p* < 0.01) are substantially larger than the main effect coefficient of organizational politics perception (M1: POP, γ =  − 0.107, *p* < 0.01). This finding warrants further discussion. However, an examination of coefficient magnitudes indicates that under circumstances characterized by elevated political skill and substantial environmental uncertainty (Table 4: the projected EIB value for the high PS, high EU cohort when POP is high is 4.543, which is demonstrably greater than the low POP group value of 3.537), a high POP is associated with enhanced innovative performance among employees possessing high political skill and operating within highly uncertain environments. This observation implies that political acumen might enable individuals to leverage perception of organizational politics as avenues for securing resources and fostering idea generation. In other words, in specific contexts, political skill may offset the negative impacts of POP and even generate positive behavioral outcomes, rather than merely buffering negative effects. This finding extends prior research’s simplistic understanding of political skill as a mere “buffer” and suggests that future studies should further explore whether political skill can produce a “reversal effect” and the boundary conditions under which it occurs. To some extent, the results are expected, as extant research shows that political skill is a moderator in negative situations^38^, and they are consistent with and further develop the research of Zhou and Wu, Malik et al., Chen et al. and Kaur and Kang^19–21,38^.

### Theoretical implications

First, this study focuses on employee innovative behavior rather than innovation capability. It develops a cross-level integrated framework to unpack the joint moderating effect of individual-level political skill and organizational-level environmental uncertainty on the POP–EIB relationship. This responds to repeated scholarly calls to explore how individual traits and situational factors interact to shape workplace innovation outcomes^50^, while filling the cross-level research gap regarding inhibitory antecedents of EIB. Much of the existing literature examines individual or organizational factors in isolation and fails to fully explain how individual cognition interacts with organizational contexts to influence innovative behavior^12^. By incorporating individual political skill and organizational environmental uncertainty into a single model, this study identifies cross-level boundary conditions for the POP–EIB linkage and offers new insights into factors that constrain EIB.

Second, this study advances research on organizational politics in the Chinese cultural context and deepens understanding of the functional mechanisms of political skill. Shaped by Confucian values, Chinese workplaces are characterized by high power distance, strong hierarchical awareness, and a relationship-oriented culture, making organizational politics a widespread phenomenon across domestic firms^5,6^. While prior work has validated the negative influence of perception of organizational politics on innovation^19,20^, limited research has examined whether and how political skill alleviates such adverse impacts within China’s distinct socio-political environment. The present study verifies that political skill negatively moderates the POP–EIB relationship. This finding extends the application of political skill theories in innovation research, enriches boundary condition studies in organizational politics research, and lays a theoretical groundwork for mitigating the harmful outcomes of workplace politics.

Third, this study provides empirical evidence for reciprocal determinism and broadens research perspectives on environmental uncertainty within the innovation literature. Reciprocal determinism posits that individual cognition, behavior and the environment interact to jointly shape human conduct^52^. Similarly, social cognitive theory and social information processing theory contend that individuals adjust their attitudes and behaviors in response to environmental cues^27^. This research integrates individual political perceptions, political skill and organizational environmental uncertainty into one analytical model. The results confirm that environmental uncertainty amplifies the buffering effect of political skill against the negative impacts of POP. Overall, our findings elaborate the interactive dynamics between individual characteristics.

### Practical implications

In the Chinese cultural context, this study provides a theoretical direction and practical explanation for enterprise managers to cope with organizational politics, enhance employees’ innovative behavior, and summarize local management experience. On the one hand, the POP is the result of the lack of a fair and reasonable organizational environment. It reduces the effort made by staff and hinders the promotion of innovative behavior. Although organizational political behavior is difficult to avoid, managers can reduce POP by establishing transparent rules and fair operational procedures, ensuring a fair resource allocation process, implementing transparent performance appraisal systems and assessment and encouraging employees to speak up. This will enhance employees’ perceived fairness, closely tie organizational incentives, rewards, and punishments to work performance, and restrict and guide organizational political behaviors outside formal rules and regulations to mitigate the negative influence of the POP.

Innovation compels organizations to adapt to dynamic conditions within a complex socio-political landscape, particularly amid today’s highly uncertain environment. For both managers and employees, political skill is a key factor enabling survival and growth amid ongoing environmental shifts. Accordingly, organizational members should recognize the functional value of political skill in workplace settings. Organizations may also deliver targeted training to nurture such competencies, so as to fully leverage their potential for boosting innovation.

Even so, several caveats need to be noted. Our results confirm that political skill buffers the detrimental influence of POP on EIB, indicating that firms can mitigate the harms of politicized environments by developing employees’ political capabilities. That said, large-scale, overt training focused on political skill may bring about unintended outcomes. An overemphasis on political skill could drive employees to prioritize power struggles and interest bargaining, which in turn exacerbates POP. Some employees may even exploit learned political skills to pursue self-interested aims, further deteriorating the overall political climate.

For this reason, alongside individual political skill development, organizations must prioritize institutional measures to depoliticize the workplace. For example, transparent and fair performance appraisal and resource allocation systems, together with reduced information asymmetry and ambiguous regulations, can fundamentally curb the emergence of organizational politics. In short, political skill training is merely a remedial solution. The fundamental approach lies in restraining organizational politics via institutional improvement.

Against the backdrop of digital transformation, firms can facilitate EIB by boosting employees’ engagement in digital learning—covering behavioral, affective and intellectual dimensions—and cultivating an innovation-supportive organizational climate^53^. Specifically, offering structured digital learning resources and encouraging knowledge sharing helps employees translate learning gains into tangible innovative behaviors.

### Limitations and research prospects

This research has certain limitations that should be addressed in future research. First, several scales were originally developed in Western contexts. Organizational politics and related behaviors may manifest differently across cultural settings in terms of political behavior, political skill, and innovative behavior. Future studies may revise Western scales or develop indigenous measurement tools tailored to the Chinese context so that the variable measurement and research results can reflect the reality of China more closely.

Second, this study adopted a questionnaire survey to collect self-reported data from employees across various industries in China. The independent variable (POP), moderating variables (PS and EU), and dependent variable (EIB) were all measured via employee self-reports. While we adopted a two-wave time-lagged design to alleviate common method bias, same-source bias could still exist. To address this limitation, future studies may collect leader–employee dyadic data, with immediate supervisors rating their subordinates’ innovative behavior.

In addition, the POP scale used in this study was developed and revised by Ma et al.^45^ based on Chinese samples and has obtained partial empirical validation in prior domestic research. Nevertheless, workplace political behaviors in China carry unique cultural traits, which cannot be fully captured by scales originating from Western contexts. Although our scale covers items related to fairness and resource allocation (e.g., “In our organization, people need to carefully consider whom not to offend”), it does not include dedicated items measuring indigenous constructs such as face and clique culture. Accordingly, future research could develop culturally indigenous scales for organizational politics perceptions or adopt qualitative approaches such as in-depth interviews to better unpack the unique manifestations of political behaviors in Chinese organizations.

Furthermore, EIB and its predictors may differ across nations, regions, industries and organizational cultures. Cross-cultural and cross-industry comparisons combining subjective and objective data will help verify the generalizability of our findings beyond the Chinese setting. It is also worthwhile for future research to examine potential mediating mechanisms underlying the focal relationships, so as to advance theories on organizational politics and innovation.

This study solely relies on quantitative empirical methods, which limits our ability to explore in-depth mechanisms linking POP to EIB within real organizations. Future work could select typical enterprise cases and conduct ethnographic research and grounded theory analysis. Such efforts can further reveal underlying behavioral mechanisms and strengthen the theoretical and practical implications of the present results.

Moreover, we examined the effects of POP on EIB as well as its boundary conditions using cross-sectional data. It remains unclear whether this relationship varies across different developmental stages of organizations. From a developmental perspective, longitudinal tracking and mixed-method designs are recommended for future research to explore how the POP–EIB relationship evolves and differs throughout organizational development.

Notably, key cultural factors including Confucian values, mianzi (face) and guanxi were not empirically measured in this study. Accordingly, our cultural interpretations remain tentative and call for follow-up empirical tests via cross-cultural and indigenous research designs. Future research could also address the limitations of this study.

## Supplementary Information

Below is the link to the electronic supplementary material.


Supplementary Material 1


## Data Availability

All data generated or analysed during this study are included in our Manuscript and Supplementary Information files. The data and scale items supporting the findings of this study are available from the corresponding author (20220075@email.sau.edu.cn) upon reasonable request.
