# Supplementary material for: Perceived organizational politics and employees’ innovative behavior: a cross-level joint moderation model
Source: Sci Rep. 2026 Jul 6;16:27391. doi: 10.1038/s41598-026-59802-2 (PMC13534711; doi:10.1038/s41598-026-59802-2)
Supplement: Supplementary file 1 [file 41598_2026_59802_MOESM1_ESM.docx]

#### How the perception of organizational politics affects employees’ innovative behavior: A cross-level model of joint moderating effect

#### Xiaona Xu^1,*^,Jingxue Wang^2^,Yan Liu^3^

#### E-mail: [20220075@email.sau.edu.cn](mailto:20220075@email.sau.edu.cn)

#### ^1,2,3^School of Economics and Management, Shenyang Aerospace University,

#### Shenyang, Liaoning, China.

#### The preprint paper mentioned in the references

Na, Y., Lu, Y., Wang, Y., & Yi, L. (2026). How challenge demands affect employee innovative behavior? The dual role of team leaders’ Dark Triad personality traits. *Journal of Business Research, 204,* 115851. <https://doi.org/10.1016/j.jbusres.2025.115851>.

**Questionnaire**

| Variables and measurements | Loadings |
| --- | --- |
| **PERCEPTION OF ORGANIZATIONAL POLITICS** |  |
| **Self-interest behavior** |  |
| In (our) organization, there are certain small groups that have the advantage of resource acquisition and communication. | 0.784 |
| In (our) organization, some people or groups can do whatever or get everything they want if you find the right person. | 0.795 |
| In (our) organization, people should think carefully about who they can't go against. | 0.812 |
| In (our) organization, managers use various policies to serve their own development. | 0.777 |
| In (our) organization, the squeaky wheel gets the grease. | 0.814 |
| In (our) organization, it's usually favored rather than talented people who rise to the top. | 0.812 |
| In (our) organization, it is usually only a few people who benefit from policy changes. | 0.811 |
| **Pay and promotion** |  |
| In (our) organization, employees who do a good job are often promoted. | 0.792 |
| In (our) organization, employees who work hard are rewarded. | 0.806 |
| In (our) organization, pay and promotion standards are well established. | 0.789 |
| In (our) organization, employees are well aware of the organization's compensation and promotion policies. | 0.807 |
| In (our) organization, leaders encourage employees to speak up, even when criticizing authority views. | 0.761 |
| **Relationship between colleagues** |  |
| In (our) organization, people only care about or do things that are relevant to them. | 0.872 |
| In (our) organization, colleagues only lend a helping hand when there is a benefit. | 0.858 |
| In (our) organization, managers are perceived as helping others only on the surface. | 0.860 |
| In (our) organization, managers communicate with their subordinates mainly to improve their own image. | 0.861 |
| **POLITICAL SKILL** |  |
| **Networking ability** |  |
| I spend a lot of time and effort at work networking with others. | 0.807 |
| At work, I know a lot of important people and am well connected. | 0.760 |
| I am good at using my connections and networks to make things happen at work. | 0.776 |
| I have developed a large network of colleagues and associates at work who I can call on for support when I really need to get things done. | 0.795 |
| I spend a lot of time at work developing connections with others. | 0.764 |
| I am good at building relationships with influential people at work. | 0.834 |
| **Apparent sincerity** |  |
| It is important that people believe I am sincere in what I say and do. | 0.872 |
| When communicating with others, I try to be genuine in what I say and do. | 0.839 |
| I try to show a genuine interest in other people. | 0.878 |
| **Social astuteness** |  |
| I always seem to instinctively know the right thing to say or do to influence others. | 0.798 |
| I have good intuition or savvy about how to present myself to others. | 0.853 |
| I am particularly good at sensing the motivations and hidden agendas of others. | 0.778 |
| I pay close attention to people’s facial expressions. | 0.822 |
| I understand people very well. | 0.787 |
| **Interpersonal influence** |  |
| It is easy for me to develop good rapport with most people. | 0.813 |
| I am able to make most people feel comfortable and at ease around me. | 0.866 |
| I am able to communicate easily and effectively with others. | 0.793 |
| I am good at getting people to like me. | 0.795 |
| **ENVIRONMENTAL UNCERTAINTY** |  |
| **Technical uncertainty** |  |
| The speed of technology updates in the industry is very fast. | 0.860 |
| Technological change in the industry is difficult to predict. | 0.846 |
| The complexity of technology in the industry is very high. | 0.866 |
| **Market uncertainty** |  |
| The market in the industry is rapidly changing. | 0.834 |
| The market in the industry is difficult to predict. | 0.766 |
| The complexity of the market in the industry is very high. | 0.793 |
| **EMPLOYEES’ INNOVATIVE BEHAVIOR** |  |
| I search out new technologies, processes, techniques, and/or product ideas. | 0.892 |
| I generate creative ideas. | 0.731 |
| I promote and champion ideas to others. | 0.821 |
| I investigate and secure funds needed to implement new ideas. | 0.755 |
| I develop adequate plans and schedules for the implementation of new ideas. | 0.815 |
| I am innovative. | 0.803 |

**Datasets**
